# Total versus staged versus functional revascularization in NSTEACS patients with multivessel disease

**DOI:** 10.1186/s43044-021-00179-0

**Published:** 2021-06-26

**Authors:** Ahmed O. Elkady, Mohamed Abdelghany, Reda Diab, Ahmed Ezz, Abdalla A. Elagha

**Affiliations:** 1 Cardiology Department, Kobry El-kobba Military Medical Hospital, Cairo, Egypt; 2 Cardiovascular Department, Kasr-Alainy Hospital, Cairo University, 1 Saraya St., Third Floor, Manial, Cairo, Egypt

**Keywords:** Non-ST segment elevation acute coronary syndrome (NSTEACS), Acute coronary syndrome (ACS), Fractional flow reserve (FFR)

## Abstract

**Background:**

The optimal strategy for revascularization in patients with NSTEACS who had multivessel coronary artery disease. A lack of evidence exists about the role of complete coronary revascularization by PCI in patients with non-ST segment elevation acute coronary syndrome (NSTEACS). Till now, ACC/AHA and ESC guidelines are not clear regarding the optimal strategy for revascularization in NSTEACS patients with multivessel coronary artery disease. In this setting, identification of the culprit lesion by angiography only could be challenging. The objective is to compare the hospital and short-term (6 months) outcomes of 3 different coronary revascularization strategies in NSTEACS patients with and multivessel coronary artery disease.

**Results:**

Our study was a prospective study that included 90 patients who presented with acute chest pain and were diagnosed with NSTEACS. The patients were divided into 3 groups according to the plan of management: total revascularization group (total group), staged revascularization group (staged group), and functional revascularization group using FFR (FFR group). We studied the effect of demographic data, risk factors, and angiographic and procedural criteria on hospital and short-term outcomes. No significant statistical difference was seen among the three groups regarding the hospital outcome (in-stent thrombosis, unstable angina, and renal impairment). Also, the short-term (after 6 months) outcome regarding myocardial infarction, hospitalization, stroke, and cardiac death did not differ significantly between the three groups.

**Conclusions:**

Considering NSTEACS patients with multivessel disease, different coronary revascularization strategies (total, staged, or FFR) are comparable regarding immediate and short-term (6 months) clinical follow-up. FFR can change the preplanned management, and less number of stents per patient is needed when FFR is utilized.

## Background

Visual assessment of the severity of coronary artery disease in non-ST segment elevation acute coronary syndrome (NSTEACS) patients is usually the guide that directs the management decision either medical treatment, percutaneous coronary intervention (PCI), or coronary artery bypass surgery (CABG) [1, 2]. This assessment of lesion severity utilizing coronary angiography by cardiologists may be inaccurate resulting in under- or overestimation of the physiological significance of the lesion. Frankly speaking, judgments made by interventional cardiologists in everyday practice are frequently subjective and potentially can lead to misdiagnosis and incorrect management strategy [3, 4]. Identification of the culprit lesion in the setting of NSTEACS by angiography alone could be challenging. Secondary plaque ruptures in patients with ACS are frequent (about 25%) as suggested by histopathological, intravascular ultrasound, and optical coherence tomography analysis. A benefit for multivessel PCI in patients with STEMI and multivessel disease has been suggested by multiple randomized trials [5, 6]. On the other hand, the role of single versus staged PCI in NSTEACS was studied by only one dedicated trial [7].

To our knowledge, this is the first study to compare total versus staged versus functional coronary revascularization in NSTEACS patients.

## Methods

### Patient selection

This prospective study was conducted on 90 patients at the Kobry El-kobba Military Hospital during a 12-month period. The inclusion criteria were as follows: diagnosis of NSTEACS according to the ESC 2015 guidelines, coronary angiography showing multivessel disease, creatinine clearance > 60 ml/min, planned early invasive strategy within 48 h from the presentation, and signed informed consent. The exclusion criteria were as follows: cardiogenic shock, chronic total occlusion, previous coronary artery bypass graft surgery, SYNTAX score > 32, indication for bypass surgery (severe valvular disease, left main disease), patients with STEMI, previous history of anaphylactoid reaction to contrast media, acute renal failure or severe chronic non-dialysis-dependent kidney disease, and patients who refused to provide consent for study enrollment.

### Study design

After coronary angiography was done and interpreted by at least two experienced interventional cardiologists, the patient was randomly assigned to one of the three management plans using “1:1:1 randomization method.” The three management plans are as follows: (1) total revascularization (total group) in which all diseased coronaries would be completely revascularized during the index procedure, (2) staged revascularization (staged group) in which only the culprit artery would be revascularized during the index procedure followed by complete coronary revascularization of non-culprit lesions in another session within the following 6 weeks, and (3) functional revascularization (FFR group) in which revascularization would be performed only to the hemodynamically significant lesions determined by FFR.

The culprit vessel was determined using ECG, echocardiography, or/and angiography criteria. An FFR ≤ 0.80 is an evidence-based functional threshold that correlates with the presence of a hemodynamically significant lesion, while values > 0.80 indicate that patients can be managed safely with medical therapy without the need for coronary stenting.

Procedural data (duration, contrast volume, complication, and success rate) were documented for each patient. Also, hospital course (duration of hospitalization, adverse events, and primary outcome) was obtained. Furthermore, short-term outcomes via office-based direct visits were performed at 1 and 6 months.

According to the presence or absence of elevated cardiac biomarkers, our patients were divided into the non-ST elevation myocardial infarction (NSTEMI) group (patients who had elevated cardiac troponin) and unstable angina (UA) group (patients with normal troponin).

## Results

Male gender was more prevalent in our study (74 male and 16 female patients). The mean age was 61.22 ± 7.52 years. All our patients presented with NSTEACS; 17 patients had signs of lung congestion. Fifty-five patients were found to be hypertensive, 61 patients were recorded as active smokers, 39 patients were diabetics, and 35 patients had a positive family history of premature coronary artery disease. Sixty-three patients were found to have dynamic ECG changes at the time of presentation. Twenty-six of our patients had positive troponin. Regarding echocardiography, the mean EF was 54.71% ± 6.23%.

### Procedure data and outcome

The mean duration of the procedure was 41.08 ± 13.75 min, the mean volume of contrast used was 300.56 ± 84.63 ml, and the mean value of the SYNTAX score was 11.31 ± 3.03. Regarding the outcome, nine of our patients suffered chest pain during the hospital stay, and they were all managed medically. Only one patient developed renal impairment, and no one developed in-stent thrombosis (manifested by ECG and cardiac enzyme abnormalities).

### Follow-up

**Fig. 1:**
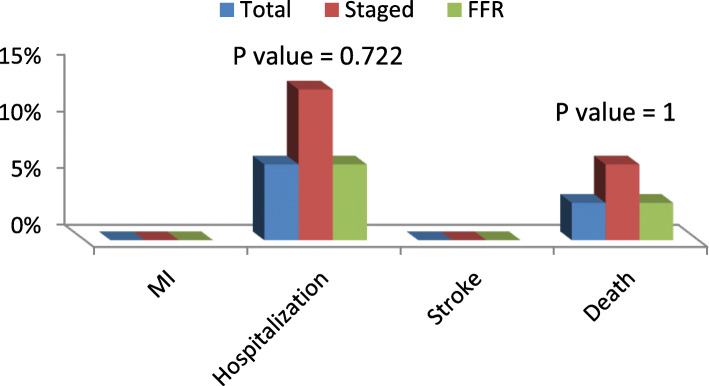
The hospital outcome in the three groups

Eight patients were re-hospitalized, and 4 patients suffered cardiac death during the 6-month follow-up period (Fig. 1). No myocardial infarction or cerebrovascular events were encountered during the follow-up period (Table 1).

**Table 1:** The hospital outcome of the three groups. After the procedure was finished, the patient was put under observation for 3–5 days. There was only one patient who suffered renal impairment from the total group. Also, there were 5 patients from the staged group who experienced unstable angina during the hospital stay that was compared to 2 patients and only one patient in the total and FFR groups, respectively

	Total		Staged		FFR		P value
	No. of patients	%	No. of patients	%	No. of patients	%	
MI	0	0.0%	0	0.0%	0	0.0%	–
Hospitalization	2	6.7%	4	13.3%	2	6.7%	0.722
Stroke	0	0.0%	0	0.0%	0	0.0%	–
Death	1	3.3%	2	6.7%	1	3.3%	1
Total	3	10%	6	20%	3	10%	0.578

Comparison among the three groups: total revascularization (total group), staged revascularization (staged group), and functional revascularization (FFR group), was done regarding all the recorded data. The mean age was comparable among the 3 groups (P value = NS). There was no statistically significant difference between the three groups regarding the clinical presentation of patients (P value = 0.356). The subjects of the three groups had comparable results regarding their associated risk factors (hypertension, diabetes, smoking, and positive family history of premature coronary artery disease). The distribution of dynamic ECG changes among the patients of the three groups was comparable (P value = 0.263). Positive troponin was found in 8 patients in both the total and the FFR groups and 10 patients in the staged group; however, there was no statistically significant difference between the three groups (P value = 0.805). The mean EF was comparable among the three groups (P value = 0.546).

Regarding the angiographic criteria, the LAD was the most affected vessel among the three groups, and this was similar in the three groups. The mean value of the SYNTAX score was comparable between the three groups (P value = 0.736).

Although multivessel disease was an inclusion criterion, however, the FFR changed the decision in 7 patients (23.33%) who had only one vessel revascularized (hemodynamically significant lesion FFR ≤ 0.80), and this difference was statistically significant (P value = 0.001). As shown in Table 2, there is no “one vessel” only revascularization in the total and staged groups (multivessel disease was an inclusion criterion); however, there were 29 patients that represents 96.63% of the total group patients who had two vessels revascularized and only one patient had three vessels revascularized. On the other hand, there were only two patients in the staged group who had a total of three vessels revascularized (all PCI settings). However, there were 23 patients in the FFR group who had two-vessel revascularization (no patient had three-vessel revascularization in the FFR group).

**Table 2:** The short-term outcome of the three groups. After 6 months, there was an office- or/and telephone-based interview done with each patient to evaluate the short-term outcome of the management plan. Two patients in both the total and the FFR groups were hospitalized due to unstable angina, and this was compared to 4 patients in the staged group. The staged group had the greatest incidence of complication between the three groups as there were 6 patients (20%) who suffered complications during this period

		Total		Staged		FFR		P value
		Count	Col %	Count	%	Count	%	
Treated vessel	Single vessel	0	0%	0	0%	7	23.33%	0.001
	Two vessels	29	96.67%	28	93.33%	23	76.67%	0.070
	Three vessels	1	3.33%	2	6.67%	0	0%	0.770

Also, the total number of stents used was 73 and 72 in the total and the staged groups, respectively, compared to only 61 stents used in the FFR group. This difference was statistically significant (P value = < 0.001). The duration of the procedure and the volume of contrast used were the least in the staged group, which was statistically significant (P value = < 0.001).

**Fig. 2:**
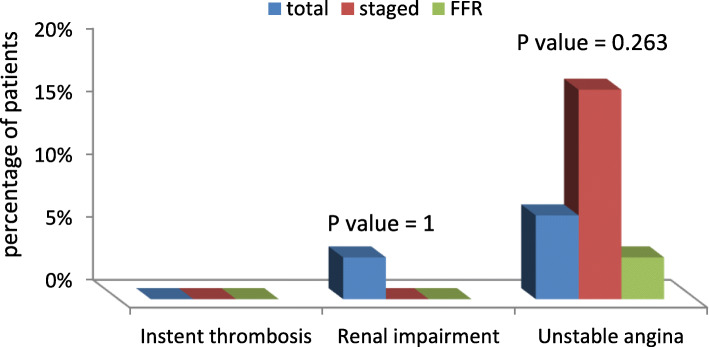
The short-term outcome in the three groups

There was only one patient who suffered renal impairment from the total group, and he was managed medically that did not require dialysis (Table 3). Also, there were 5 patients (16.67%) from the staged group who experienced anginal pain during the hospital stay that was compared to 2 patients in the total group and only one patient in the FFR group. However, no in-stent thrombosis complication was encountered among the three groups (Fig. 2). Finally, the incidence of major adverse cardiac and cerebrovascular events was comparable among the three groups.

**Table 3:** Short-term complications. There was only one patient who suffered renal impairment from the total group. Also, there were 5 patients (16.67%) from the staged group who experienced anginal pain during the hospital stay that was compared to 2 patients in the total group and only one patient in the FFR group

	Total		Staged		FFR		P value
	No.	Col %	No.	%	No.	%	
In-stent thrombosis	0	0.0%	0	0.0%	0	0%	–
Renal impairment	1	3.3%	0	0.0%	0	0%	1
Unstable angina	2	6.67%	5	16.67%	1	3.33%	0.263
Total	3	10%	5	16.67%	1	3.33%	0.284

### NSTEMI versus unstable angina

Twenty-six of our patients had elevated troponin (NSTEMI), and 64 were diagnosed as (UA) patients.

Eight NSTEMI patients were in the total revascularization group, 8 NSTEMI patients were in the FFR guided revascularization group, and 10 NSTEMI patients were in the staged revascularization group.

We found no statistically significant difference between the three groups in the distribution of NSTEMI and UA (p = NS).

Also, there was no statistically significant impact on the outcomes of patients (p = NS).

## Discussion

The main finding of our study is that in NSTEACS patients with multivessel disease, coronary revascularization strategies (total, staged, or FFR guided) are comparable regarding immediate and short-term (6 months) clinical follow-up.

We included ninety patients in our study who presented to the ER with NSTEACS in our hospital, in an 8-month period. Being performed in a military medical hospital may explain the predominance of the male gender among the study population. The similarity of basic characteristics and laboratory findings of patients in the three groups (regarding demographic criteria, risk factors, clinical presentation, ECG, echocardiography, laboratory, and angiographic findings) allows for a better comparison among the three groups.

Indeed, our study protocol was similar to the FAMOUS-NSTEMI trial, which found that FFR disclosure resulted in a change in the treatment plan of 144 patients planned for PCI. After applying FFR, only 117 patients underwent PCI [8]. This could be explained by the fact that measuring FFR changes the classification of stenosis from “significant stenosis” for PCI to “non-significant stenosis” managed by only optimal medical treatment. In agreement with the results of the FAME trial, we found that significantly less stents per patient were placed in the FFR group than in the other groups [9]. Furthermore, the 2-year follow-up of the FAME Study found that the number of stents used was 2.7 ± 1.2 in the angiography-guided group and 1.9 ± 1.3 in the FFR-guided group (p < 0.001) [10]. This is explained simply due to less number of lesions indicated for PCI after measuring FFR.

Our study found that the volume of contrast used was least in the staged group than that used in the total and FFR groups. In agreement with our results, the “CvLPRIT” trial demonstrated that total contrast used was 250 (190–330) ml in the “complete revascularization group” versus 190 (150–250) ml in the “infarct-related artery (IRA)-only revascularization group” (P value < 0.0001) [6].

Regarding the mean procedure, we found that duration was least in the staged group than in the total and FFR groups. Similarly, in the CvLPRIT trial, the total procedure time was 55 (38–74) min in the “complete” revascularization group, while in the “infarct-related artery (IRA)-only revascularization group,” it was 41 (30–55.5) min (P value < 0.0001) [6]. This can be explained by the fact that in the staged group, the duration of the procedure and volume of contrast needed to deploy stent(s) in only one vessel are significantly lower than that needed to fix all stenosed vessels in the total group or all the functionally significant lesions in the FFR group.

Regarding the procedure outcome, our study found that there was no statistically significant difference between the three groups. In concordance with our results, the FAMOUS-NSTEMI trial found that in-hospital adverse events were similar in the “FFR-disclosure group” and “angiography group” [8]. Also, the “CvLPRIT trial” found that the periprocedural events and ischemia testing were similar in the complete revascularization and IRA-only revascularization groups [6]. In contrast to our results, the “FAME Study” found that hospital stay at baseline admission was significantly more in the angiography group than that in the FFR group [10].

We found that major acute cerebrovascular and cardiac events were comparable among the three groups at the 6-month follow-up. In agreement with our results, the FAMOUS-NSTEMI trial found that 14 (8.0%) of 176 patients in the “FFR-guided group” and 15 (8.6%) of 174 in the “angiography-guided group” experienced cardiac death, non-fatal myocardial infarction, or heart failure hospitalization (P = 0.89). Myocardial infarction relating to revascularization occurred in 5 (2.8%) patients in the “FFR-guided group” and 11 (6.3%) patients in the “angiography-guided group” (P = 0.12). Major adverse cardiac events excluding MI related to revascularization occurred in 10 (5.7%) patients in the “FFR-guided group” and 5 (2.9%) patients in the “angiography-guided group” (P = 0.25) [8]. Also supporting our results, the FAME Study showed that all-cause mortality at 2 years was 3.8% (n = 19) in the angiography-guided group and 2.6% (n = 13) in the FFR-guided group (p = 0.25). After 2 years, MACE had occurred in 111 patients (22.4%) in the angiography-guided group and in 91 patients (17.9%) in the FFR group (p = 0.08) [10]. According to the results of our study, there was no statistically significant difference between the three groups in the hospital outcome or in the short-term outcome.

In contrast to our results, the major findings of “SMILE trial” were as follows: “one-stage” complete coronary revascularization is superior to “multistage” complete coronary revascularization in terms of MACE [7]. Similarly, the “CvLPRIT trial” found that MACE was significantly lower in the complete revascularization arm (10.0%) than in the IRA-only arm (21.2%) [6]. Another study observed that complete coronary revascularization is associated with a lower rate of the composite endpoint (death, myocardial infarction, or revascularization) [11].

Interestingly, we found that the FFR-guided approach resulted in changes in stenosis classification and patient management in 23.33% (n = 7) of the FFR group patients. The rate of coronary revascularization was reduced at the index procedure, and this difference was maintained at 6 months.

Finally, we believe that a larger and longer study may be necessary to properly assess the differences between the three management plans regarding health outcomes and cost-effectiveness.

### Limitations

A small sample size that is limited only to a single center was the most prominent limitation. A short-term follow-up period was implemented; however, a longer period is needed to detect all possible health care outcomes. A cost-benefit study of each management plan was not performed, which may help to set management policies in developing countries with limited resources.

## Conclusion

Considering NSTEACS patients with multivessel disease, different coronary revascularization strategies (total, staged, or FFR) are comparable regarding immediate and short-term (6 months) clinical follow-up. FFR can change the preplanned management, and less number of stents per patient is needed when FFR is utilized. We believe that a larger study with a longer follow-up period is needed to determine the optimal strategy for NSTEACS patients with multivessel disease.

## Data Availability

All data generated or analyzed during this study are included in this published article.

## Abbreviations

CABG: Coronary artery bypass surgery
FFR: Fractional flow reserve
IRA: Infarct-related artery
LAD: Left anterior descending
NS: Not significant
NSTEACS: Non-ST segment elevation acute coronary syndrome
PCI: Percutaneous coronary intervention
